# Feasibility and safety of mass drug coadministration with azithromycin and ivermectin for the control of neglected tropical diseases: a single-arm intervention trial

**DOI:** 10.1016/S2214-109X(18)30397-8

**Published:** 2018-09-14

**Authors:** Lucia Romani, Michael Marks, Oliver Sokana, Titus Nasi, Bakaai Kamoriki, Handan Wand, Margot J Whitfeld, Daniel Engelman, Anthony W Solomon, Andrew C Steer, John M Kaldor

**Affiliations:** a The Kirby Institute, University of New South Wales, Sydney, NSW, Australia; b Murdoch Children's Research Institute, Melbourne, VIC, Australia; c Clinical Research Department, Faculty of Infectious & Tropical Diseases, London School of Hygiene & Tropical Medicine, London, UK; d Hospital for Tropical Diseases, London, UK; e Ministry of Health and Medical Services, Honiara, Solomon Islands; f St Vincent's Hospital Sydney, Sydney, NSW, Australia; g Centre for International Child Health, University of Melbourne, Melbourne, VIC, Australia; h Department of Control of Neglected Tropical Diseases, World Health Organization, Geneva, Switzerland

## Abstract

**Background:**

Mass drug administration has made a major contribution to the public health control of several important neglected tropical diseases. For settings with more than one endemic disease, combined mass drug administration has potential practical advantages compared with separate programmes but needs confirmation of feasibility and safety. We undertook a study of mass drug administration in the Solomon Islands for trachoma and scabies control using ivermectin and azithromycin, key drugs in the control of neglected tropical diseases worldwide.

**Methods:**

The entire population of Choiseul province, Solomon Islands, was eligible to participate. An azithromycin-based mass drug administration regimen was offered in line with standard recommendations for trachoma elimination (oral azithromycin or topical tetracycline). An ivermectin-based mass drug administration regimen was offered at the same time (oral ivermectin or topical permethrin), with a further dose 7–14 days later, using a modified version of a regimen demonstrated to be effective for scabies control. All participants underwent safety assessments 7–14 days later. Participants in ten randomly selected sentinel villages underwent a more detailed safety assessment. Routine health system reports of hospital or clinic admissions and deaths were also obtained to compare health outcomes in the 12 month period before and after the mass drug administration.

**Findings:**

The study enrolled 26 188 participants, 99·3% of the estimated resident population as determined at the 2009 census. Of those enrolled, 25 717 (98·2%) received the trachoma regimen and 25 819 (98·6%) received the first dose of the scabies regimen between Sept 1, and Oct 2, 2015. A second dose of the scabies regimen was received by 21 931 (83·7%) of participants. Adverse events, all mild and transient, were recorded in 571 (2·6%) of the entire study population and 58 (4·1%) of participants in the ten sentinel villages. In the 12 months before and after the mass drug administration the numbers of hospital admissions (1530 *vs* 1602) and deaths (73 *vs* 83) were similar. In the month after the mass drug administration, 84 individuals were admitted to hospital and two died, compared with a monthly median of 116 admissions (IQR 106–159) and six deaths (IQR 4–7) in the 12 months before and after the mass drug administration.

**Interpretation:**

In the largest trial so far involving coadministration of regimens based on ivermectin and azithromycin, the combination was safe and feasible in a population of more than 26 000 people. Coadministration of mass drug administration based on these two drugs opens up new potential for the control of neglected tropical diseases.

**Funding:**

International Trachoma Initiative, Murdoch Children's Research Institute, Scobie and Claire Mackinnon Trust, Wellcome Trust.

## Introduction

Mass drug administration involves treatment of whole populations with a pharmaceutical agent to reduce or interrupt transmission of an infectious pathogen. It has become a key strategy for the control of neglected tropical diseases in several low-income and middle-income countries.^1^ The five main neglected tropical diseases currently targeted by mass drug administration are onchocerciasis, lymphatic filariasis, trachoma, schistosomiasis, and infection with soil-transmitted helminths, diseases that are strongly related to living conditions and primarily affect rural populations in resource-limited settings.^2^, ^3^ Mass drug administration is intended to complement improvements in both environmental risk factors and health service access, to ultimately achieve elimination of these diseases.^4^

Many communities are affected by multiple neglected tropical diseases.^5^, ^6^ Although some programmes for mass drug administration target more than one neglected tropical disease (eg, combined ivermectin and albendazole for both lymphatic filariasis and onchocerciasis), they are more often aimed at single diseases. There has been increasing international recognition of the need to better integrate these vertical structures, both with each other, and with local health systems.^7^, ^8^, ^9^, ^10^ The potential logistical and health benefits of integration include cost savings, reduced burden on health systems and communities, and better disease control through improved coverage. However, uncertainties remain about the feasibility of integration in resource-limited settings, and about the efficacy and safety of coadministration of drugs on a large scale.^1^, ^7^, ^11^

**Research in context**

**Evidence before this study**

We searched PubMed, Ovid Medline, and Embase, without language restrictions, for studies reporting on the mass coadministration of azithromycin-based and ivermectin-based regimens, published up to Nov 15, 2017, with the terms “azithromycin” and “ivermectin”. We identified one study of 1500 participants in a setting of endemic neglected tropical disease, showing that this combination (plus albendazole) resulted in mild, transitory adverse events consistent with the known profiles of the individual drugs when used alone, and no evidence of potentiation. This study, however, provided insufficient power to detect uncommon adverse events arising from coadministration and highlighted the need for large field studies of this strategy. Additionally, it did not investigate coverage and feasibility issues of coadministration of these two therapies.

**Added value of this study**

Our trial was designed to investigate the feasibility and safety of mass drug administration of two integrated therapies with azithromycin and ivermectin. To our knowledge, it is the first published large-scale trial of coadministration of this strategy to control neglected tropical diseases, providing safety information on more than 26 000 people as opposed to 1500 in the only previous study. In our study, safety was evaluated using active and passive monitoring of adverse events as well as a comprehensive review of routine clinic and hospital admission records for the 12 months before and after the intervention. We investigated the feasibility of joint mass administration of the two regimens, demonstrating that coadministration based on these two drugs opens up new potential for the control of neglected tropical diseases.

**Implications of all the available evidence**

The study provides evidence that coadministration of azithromycin and ivermectin is feasible and safe in a population of more than 26 000 people in a neglected tropical disease-endemic setting. We have demonstrated that a high level of population coverage is achievable in a large and geographically disperse population and provided robust evidence of the safety of this strategy in population settings. Our findings, therefore, support the strategy of integration of mass drug administration for neglected tropical diseases sharing similar target populations and therapies to reduce costs and allow a more rapid scale-up of programmes.

Azithromycin is an antibacterial drug that has been used in mass drug administration for trachoma elimination as part of the Surgery, Antibiotics, Facial Cleanliness & Environmental Improvement (SAFE) strategy for nearly 20 years.^12^ It is also effective against yaws and is recommended by WHO for this indication.^13^, ^14^, ^15^ Ivermectin is an antiparasitic drug that has been widely used for mass drug administration for lymphatic filariasis and onchocerciasis, and is also effective as mass drug administration for scabies, a newly designated neglected tropical disease.^9^, ^16^, ^17^, ^18^ With trachoma (and yaws) coendemic with combinations of lymphatic filariasis, onchocerciasis, and scabies in several countries, the joint use of azithromycin and ivermectin (with or without albendazole) might be an effective approach to improve coverage and integration of these programmes.^19^ Previous small pharmacokinetic studies of the joint administration of combinations of azithromycin, ivermectin, and albendazole in healthy volunteers have shown very few interactions.^19^, ^20^ The only previous published trial of the combination of ivermectin and azithromycin (plus albendazole) in the setting of a neglected tropical disease involved 1500 people in Mali and found mild, transitory adverse events consistent with the known profiles of the individual drugs when used alone, and no evidence of potentiation.^21^ Although promising, the study provided insufficient power to detect rare adverse events arising from coadministration and highlighted the need for large field studies of this strategy.

The Solomon Islands has high levels of both trachoma^22^, ^23^ and scabies.^24^, ^25^ On the basis of WHO SAFE strategy guidelines, mass administration of azithromycin for trachoma was implemented by the Ministry of Health and Medical Services across all ten provinces from 2014 to 2015.^22^, ^26^, ^27^ With emerging data on the effectiveness of ivermectin mass administration for scabies control,^17^ we did a trial which aimed to address outstanding questions on the feasibility and safety of large-scale coadministration of regimens based on ivermectin and azithromycin in Choiseul Province, the last province scheduled to receive mass drug administration for trachoma.

## Methods

### Study design and participants

The Azithromycin Ivermectin Mass Drug Administration (AIM) study was a prospective, single-arm, before-and-after community intervention trial to assess the feasibility and safety of joint mass drug administration with ivermectin and azithromycin for trachoma and scabies control in a large population in the Solomon Islands.

The study was done in Choiseul Province of the Solomon Islands, an island nation in the South Pacific region with a population of approximately 580 000 people across roughly 990 islands.^28^ Choiseul is located in the northwest of the country. Health care in Choiseul in 2015 was provided by 17 nurse aid posts, 10 rural health clinics, one area health centre, and a hospital located in Taro, the provincial capital.^29^

All Choiseul residents were eligible to participate. At the 2009 national census, the population of Choiseul was 26 372.^28^ The Solomon Islands National Statistics Office projected the population to be 32 548 in 2015, using an estimated annual increase in national population of approximately 2·5% per year, based on mortality, fertility, and migration rates.^30^ The projected figures are probably overestimates because they do not account for either migration to the national capital and other urban areas for school or employment, or a reported reduction in birth rates in Choiseul in recent years.^31^, ^32^

The study was approved by the Solomon Islands National Research Ethics Committee (15/33) and the Royal Children's Hospital Human Research Ethics Committee (35148A). The ethics committees approved the use of oral consent for joint administration in this study. Participants who were asked to undertake a skin examination provided written consent.

### Procedures

Drugs were delivered by Ministry teams using the procedures of the national trachoma programme, but incorporating an additional visit after 1 week to deliver the second dose of the ivermectin-based regimen for scabies control. All team members received training on study activities before the trial commencement. We obtained verbal consent, in local dialect, for individuals aged 18 years or older and verbal consent from a parent or guardian for younger individuals. Before drug administration, information sheets explaining the trial were distributed to local community nurses who were trained in study procedures, and community members were given an opportunity to meet the local health staff to ask questions. Apart from the study-specific consent process, community mobilisation efforts were identical to those that had been used by the Ministry of Health in preparing other provinces for trachoma-related mass administration of azithromycin. Individuals who consented to receive the regimen were asked to attend the village clinic or community hall to receive treatment on an agreed date.

### Interventions

The azithromycin regimen for trachoma followed WHO guidelines, identical to that used for trachoma mass drug administration in the rest of the country.^22^ Participants were offered a single oral dose of azithromycin at a dose of 20 mg/kg, using weight bands (appendix), up to a maximum of 1 g. Children weighing less than 12·5 kg received oral suspension at a dose of 20 mg/kg; others received tablets. Infants younger than 6 months were not given azithromycin, but instead were offered topical tetracycline ointment 1% for administration by a parent or guardian to both eyes twice per day for 6 weeks.

For administration of ivermectin for scabies, participants were offered a dose of oral ivermectin at 200 μg/kg, using weight bands, at the same time as the trachoma drug administration. The same dose was offered a second time, 7–14 days later. This regimen was based on a protocol shown to be safe and effective in a previous trial,^17^ with the modification that the second dose was offered to all participants (rather than being restricted to those with clinical scabies at baseline) because clinical examination of all participants was infeasible on such a large scale. Pregnant and breastfeeding women and children weighing less than 12·5 kg were offered two applications of topical permethrin cream 7–14 days apart, rather than ivermectin. We used 12·5 kg as the lower limit (rather than 15 kg, which is used for weight-based dosing of ivermectin in mass administration for lymphatic filariasis and onchocerciasis), to make administration consistent with the azithromycin weight cutoff.

Oral drug administration was directly observed for both ivermectin and azithromycin. Participants offered permethrin were given the option to apply the cream at home, or to have a trained nurse apply it in a private room at the clinic. For topical therapies (tetracycline and permethrin), appropriate use of the medication was explained on a one-to-one basis by a member of the study team to the recipient, or the recipient's carer.

### Outcomes

The coverage of mass drug administration regimens for scabies and trachoma, and of coadministration for both diseases, was calculated using the 2009 census data as the denominator, with subanalysis by age and weight bands. Coadministration was deemed successful if the coverage for both diseases was similar to that for trachoma achieved by other provinces in the same campaign (approximately 80%). For comparison with single-agent regimens, we obtained programmatic coverage data from other provinces in which the Ministry of Health had previously conducted mass administration of azithromycin for trachoma.

Safety was evaluated via four methods, incorporating standard definitions of adverse events.^33^ First, we sought information on the occurrence of immediate severe adverse events, defined as admission to hospital or death within 24 h of exposure to study medicine, as recorded in hospital records and a review of death reports. Second, we asked all participants about their current health (“Are you well today?”) at the time of initial drug administration. At the time of the second dose of the ivermectin-based scabies regimen participants were asked about their health status since the first dose (“Have you had any problem with the first dose?”). If they answered in the negative, we administered a checklist of health conditions, supplemented by free text recording of conditions not on the checklist. Third, we undertook active surveillance for adverse events in ten randomly selected sentinel villages. In these communities, we administered a questionnaire at both occasions of drug administration which sought information on the presence or absence of each condition on the checklist, again using a free text field to elicit conditions not on the list. Finally, we undertook a review of routine clinic and hospital admission records submitted through the national District Health Information System (DHIS2) during the 12 months after mass drug administration, and compared those data to data for the 12 months before mass drug administration.

### Statistical analysis

Participant characteristics were summarised by demographic categories (age, sex, and health zone) and compared with the distribution of these characteristics in the 2009 national census. We calculated coverage by age group and sex, separately for the azithromycin administration, the first dose of the ivermectin administration, both doses of the ivermectin administration, and for the full combination. All statistical analyses were done with STATA 14.0.

The trial is registered with the Australian and New Zealand Trials Registry, number ACTRN12613000474752.

## Results

26 188 people consented to participate in the study, representing 99·3% of the resident population based on the 2009 census (26 372 individuals), and 80·5% based on the 2015 projected population (32 548 individuals). The demographic features of enrolled participants were similar to those reported in the census (table 1). Delivery of mass drug administration began on Sept 1, 2015, and was completed by Oct 2, 2015.

**Table 1:** Demographic characteristics of study participants

	**Study population (n=26 188)**	**Census 2009 (n=26 372)**
**Sex**		
Male	13 259 (50·6%)	13 532 (51·3%)
Female	12 809 (48·9%)	12 840 (48·7%)
Missing	120 (0·5%)	..
**Age**		
0–4 years	3856 (14·7%)	4035 (15·3%)
5–9 years	3881 (14·8%)	3842 (14·6%)
10–14 years	3633 (13·9%)	3262 (12·4%)
15–24 years	4262 (16·3%)	4499 (17·2%)
25–34 years	3419 (13·1%)	3783 (14·3%)
≥35 years	7137 (27·3%)	6951 (26·4%)
**Zone**		
South	9225 (35·2%)	8435 (32·0%)
Northeast	5261 (20·1%)	5982 (22·7%)
Northwest	11 702 (44·7%)	11 955 (45·3%)

Of the enrolled population, 25 488 (97·3%) received the trachoma regimen and the first dose of the scabies regimen, including 21 181 (83·1%) who received both ivermectin and azithromycin at this first visit as part of these regimens. Almost all participants treated with the trachoma regimen received azithromycin (25 278 [98·3%] individuals), whereas 16·9% of participants who received the scabies regimen received permethrin rather than ivermectin (table 2). 21 817 (85·6%) participants received the trachoma regimen and both doses of the scabies regimen. 40 (0·2%) participants were not recorded as having received either regimen, and 560 (2·1%) received only one (229 [0·9%] received only treatment for trachoma and 331 [1·3%] received only treatment for scabies). Specific data on why these 560 individuals did not receive both treatments at the first visit was not available.

**Table 2:** Coverage of mass drug administration regimens for scabies and trachoma

		**Number receiving treatment (n=26 188)**
Scabies regimen first dose		25 819 (98·6%)
	Oral ivermectin	21 444 (83·1%)
	Topical permethrin	4375 (16·9%)
Scabies regimen second dose		21 931 (83·7%)
	Oral ivermectin	18 215 (83·1%)
	Topical permethrin	3716 (16·9%)
Trachoma regimen		25 717 (98·2%)
	Oral azithromycin	25 278 (98·3%)
	Topical tetracycline	439 (1·7%)
Combination scabies and trachoma regimen (first dose for scabies)		25 488 (97·3%)
Coadministration of ivermectin (first dose) and azithromycin		21 181 (83·1%)
	Combination scabies and trachoma regimen (both doses for scabies)	21 817 (85·6%)

There were no immediate serious adverse events reported. Of 21 817 participants who responded to the question on current health at the time of the second visit, 571 participants (2·62%) reported 655 adverse events since the first visit (table 3), all of which were mild and resolved within 1 week following treatment. Most commonly reported were dizziness (144 individuals, 0·7%), abdominal pain (80, 0·4%), and diarrhoea (71, 0·3%). 46 participants reported more than one event.

**Table 3:** Adverse events reported by participants

	**Adverse events overall (n=21 817)**	**Adverse events in ten sentinel villages (n=1399)**
Dizziness	144 (0·7%)	6 (0·4%)
Abdominal pain	80 (0·4%)	8 (0·6%)
Diarrhoea	71 (0·3%)	10 (0·7%)
Headache	47 (0·2%)	10 (0·7%)
Muscle pain	42 (0·2%)	4 (0·3%)
Joint pain	37 (0·2%)	5 (0·4%)
Itch	24 (0·1%)	12 (0·9%)
Nausea	15 (0·1%)	3 (0·2%)
Vomiting	6 (<0·1%)	0
Other*	59 (0·3%)	0
Individuals who experienced more than one event	46 (0·2%)	0
Individuals who experienced at least one event	571 (2·6%)	58 (4·1%)

Adverse event data were collected at the second study visit 7–14 days after the baseline coadministration of azithromycin and ivermectin.

* Other reported events included skin conditions (n=29, including 15 tinea infections and seven molluscum contagiosum), eye conditions (n=17), and other miscellaneous (n=13, including conditions such as broken knee).

Adverse events were more common in older participants (table 4). Seven children (0·2%) younger than 5 years who received ivermectin experienced adverse events. Of 1293 children weighing 12·5–15 kg who received ivermectin, seven (0·5%) experienced an adverse event. Adverse events were more frequently reported by the 21 181 participants who received azithromycin and ivermectin (513, 2·4%) compared with the 4375 who received azithromycin and permethrin (57, 1·2%, p<0·0001).

**Table 4:** Participants with adverse events by age group among those who received coadministration

	**Participants who received coadministration (n=21 817)**	**Participants with adverse events (n=571)**
0–4 years	3693	22 (0·6%)
5–9 years	3820	38 (1·0%)
10–14 years	3582	54 (1·5%)
15–24 years	4179	107 (2·6%)
25–34 years	3298	118 (3·6%)
≥35 years	6916	232 (3·4%)

In the ten sentinel villages with more detailed safety monitoring, 1399 participants were enrolled (94·6% of the registered resident population of 1479 individuals). Adverse events were reported by 58 (4·1%) participants in these villages, all mild and transient (table 3), with the most being itch (12 individuals, 0·9%), diarrhoea (ten, 0·7%), and headache (ten, 0·7%).

In the 12 months leading up to the intervention (September, 2014, to August, 2015, inclusive) there were 1530 hospital admissions and 70 deaths. In the 12 months after (October, 2015, to September, 2016, inclusive) there were 1602 admissions and 75 deaths (appendix). The median number of admissions for the entire 25 month period was 116 (IQR 106–159) and the median number of deaths was six (IQR 4–7). In October, 2015, there were 84 admissions and two deaths compared to five deaths each in September and November of the same year, respectively. These numbers did not seem to differ between the periods before and after the intervention.

## Discussion

To our knowledge, this trial is the first published report of large-scale coadministration of ivermectin and azithromycin as components of neglected tropical disease control. We have demonstrated that coadministration of the two drugs was safe and feasible. The lack of any serious adverse events in a population of more than 21 000 people, and the small number of adverse events, indicate that coadministration is a viable means of integrating programmes to control multiple, coendemic neglected tropical diseases. Furthermore, the high level of coverage achieved, both for the coadministration and the second dose of the ivermectin-based scabies regimen, indicates the feasibility of coadministration as strategy for mass drug administration. A randomised trial might have provided more detailed data on safety, but would not have been feasible on this scale, due to costs and the burden that would have been imposed on the Solomon Islands health system.

Although the benefit of mass azithromycin administration for trachoma is well established,^34^ the evidence for benefit of mass administration of ivermectin-based regimens for scabies control has emerged only recently, most notably in the SHIFT study, the strategy's first comparative trial.^17^, ^24^, ^35^, ^36^, ^37^, ^38^, ^39^ SHIFT took place in Fiji and its findings showed that a single round of mass drug administration with ivermectin reduced community prevalence of scabies by 94% at 1 year, a substantially greater reduction than either mass permethrin administration or standard care.^17^ In the present study, we have shown that a high level of population coverage can be achieved in a large and geographically dispersed population, even when including a second dose of ivermectin for the entire population. Coverage levels for the first dose (which involved joint administration of the two regimens) was similar to levels achieved for mass azithromycin administration alone in the other Solomon Islands provinces (86% in 2014–15, Oliver Sokana, personal communication). Given the high costs of transport and other logistics in remote settings, joint delivery of two drugs in mass administration regimens has the potential to deliver considerable savings. However, a specific difficulty with the current ivermectin-based regimen for scabies is the requirement of a second dose after 7–14 days, to kill newly hatched mites;^40^ this regimen is distinct from the schedules used for mass ivermectin administration for lymphatic filariasis and onchocerciasis, which require only a single dose. Although we achieved high coverage with the second dose, the logistical challenges and increased cost clearly represent a potential barrier to the wider use of ivermectin for scabies control and to routine integration with other neglected tropical disease programmes. Further studies of the efficacy of one instead of two doses for scabies, or longer-acting drugs such as moxidectin,^41^ might widen the possibilities for the integration of mass drug administration for scabies with other neglected tropical disease programmes without entailing the additional costs of delivering the second dose.

In the largest previous study, with 1500 people receiving the combination, there was no indication of clinically significant adverse events or alterations in efficacy.^21^ Our findings in a much larger population provide more robust evidence for the safety of coadministration of ivermectin and azithromycin in population settings. Our study also provides encouraging data on the safety of ivermectin in younger children, particularly those weighing as little as 12·5 kg. Gastrointestinal upset, headache, and dizziness are well recognised side-effects of azithromycin and ivermectin and were the most commonly encountered adverse events in this study, all of a mild nature.

Our study had some limitations. The design was non-randomised, so safety assessments relied on before-and-after comparisons in the same population. We also adopted a pragmatic means to assess safety across the target population. For the entire population of 26 188 participants, we sought open-ended information on health status at the time of coadministration, and again at the time of delivery of the second dose of the scabies regimen, 7–14 days after the two drugs had been received. We also sought more detailed symptom-specific information on about 6% of participants resident in the ten sentinel villages. However, we were not able to assess safety in this manner for the 3888 (16·3%) participants who did not receive the second dose. Also, we might have missed mild, transient adverse events that occurred within a short time of joint administration but had been forgotten by participants by the time they were interviewed at the second dose. We supplemented this active surveillance approach with routinely recorded data on hospital admissions and deaths in the periods preceding and following mass drug administration. The absence of any signal of increased health-care use after mass drug administration is consistent with our active surveillance data.

The integration of mass drug administration for neglected tropical diseases sharing similar target populations and drug regimens, as demonstrated in our study, has the potential to facilitate medication delivery, thereby reducing costs and allowing more rapid scale-up of programmes for multiple neglected tropical diseases. The evidence from our trial is that, on a very large scale, there were not serious adverse events arising from the joint administration of the two regimens. Studies in different populations, including different combinations of drugs (eg, triple therapy with ivermectin, albendazole, and azithromycin), are needed to further expand the evidence base for coadministration as a global strategy for control and elimination of neglected tropical diseases.

## References

[1] Hotez PJ (2009). Mass drug administration and integrated control for the world's high-prevalence neglected tropical diseases. Clin Pharmacol Ther.

[2] Hotez P, Ottesen E, Fenwick A, Molyneux D (2006). The neglected tropical diseases: the ancient afflictions of stigma and poverty and the prospects for their control and elimination. Adv Exp Med Biol.

[3] Molyneux DH, Bradley M, Hoerauf A, Kyelem D, Taylor MJ (2003). Mass drug treatment for lymphatic filariasis and onchocerciasis. Trends Parasitol.

[4] Webster JP, Gower CM, Knowles SC, Molyneux DH, Fenton A (2016). One health: an ecological and evolutionary framework for tackling neglected zoonotic diseases. Evol Appl.

[5] Dean L, Page S, Hawkins K (2016). Tailoring mass drug administration to context: implementation research is critical in achieving equitable progress in the control and elimination of helminth neglected tropical diseases in sub-Saharan Africa. Int Health.

[6] Hotez PJ (2011). The causes and impacts of neglected tropical and zoonotic diseases: opportunities for integrated intervention strategies.

[7] Emerson PM, Ngondi J, Biru E (2008). Integrating an NTD with one of “The big three”: combined malaria and trachoma survey in Amhara region of Ethiopia. PLoS Negl Trop Dis.

[8] Molyneux DH, Savioli L, Engels D (2017). Neglected tropical diseases: progress towards addressing the chronic pandemic. Lancet.

[9] Engelman D, Martin DL, Hay RJ (2013). Opportunities to investigate the effects of ivermectin mass drug administration on scabies. Parasit Vectors.

[10] Engelman D, Fuller LC, Solomon AW (2016). Opportunities for integrated control of neglected tropical diseases that affect the skin. Trends Parasitol.

[11] Molyneux DH, Hotez PJ, Fenwick A (2005). “Rapid-impact interventions”: how a policy of integrated control for Africa's neglected tropical diseases could benefit the poor. PLoS Med.

[12] Taylor HR, Burton MJ, Haddad D, West S, Wright H (2014). Trachoma. Lancet.

[13] Marks M, Vahi V, Sokana O (2015). Impact of community mass treatment with azithromycin for trachoma elimination on the prevalence of yaws. PLoS Negl Trop Dis.

[14] Mitja O, Lukehart S, Bassat Q (2016). Mass treatment with single-dose azithromycin for yaws. N Engl J Med.

[15] World Health Organization (2017). Neglected tropical diseases: scabies. http://www.who.int/neglected_diseases/diseases/scabies/en/.

[16] Cao WC, Van der Ploeg CP, Plaisier AP, van der Sluijs IJ, Habbema JD (1997). Ivermectin for the chemotherapy of bancroftian filariasis: a meta-analysis of the effect of single treatment. Trop Med Int Health.

[17] Romani L, Whitfeld MJ, Koroivueta J (2015). Mass drug administration for scabies control in a population with endemic disease. N Engl J Med.

[18] World Health Organization (2013). Neglected tropical diseases. http://www.who.int/neglected_diseases/diseases/en/.

[19] El-Tahtawy A, Glue P, Andrews EN, Mardekian J, Amsden GW, Knirsch CA (2008). The effect of azithromycin on ivermectin pharmacokinetics: a population pharmacokinetic model analysis. PLoS Negl Trop Dis.

[20] Amsden GW, Gregory TB, Michalak CA, Glue P, Knirsch CA (2007). Pharmacokinetics of azithromycin and the combination of ivermectin and albendazole when administered alone and concurrently in healthy volunteers. Am J Trop Med Hyg.

[21] Coulibaly YI, Dicko I, Keita M (2013). A cluster randomized study of the safety of integrated treatment of trachoma and lymphatic filariasis in children and adults in Sikasso, Mali. PLoS Negl Trop Dis.

[22] Sokana O, Macleod C, Jack K (2016). Mapping trachoma in the Solomon Islands: results of three baseline population-based prevalence surveys conducted with the Global Trachoma Mapping Project. Ophthalmic Epidemiol.

[23] International Agency for the Prevention of Blindness (2013). Trachoma mapping in the Pacific: Fiji, Solomon Islands and Kiribati.

[24] Lawrence G, Leafasia J, Sheridan J (2005). Control of scabies, skin sores and haematuria in children in the Solomon Islands: another role for ivermectin. Bull World Health Organ.

[25] Mason DS, Marks M, Sokana O (2016). The prevalence of scabies and impetigo in the Solomon Islands: a population-based survey. PLoS Negl Trop Dis.

[26] Solomon AW, Foster A, Mabey DC (2006). Clinical examination versus Chlamydia trachomatis assays to guide antibiotic use in trachoma control programmes. Lancet Infect Dis.

[27] Solomon AW, Zondervan ZM, Kuper H, Buchan JC, Mabey DCW, Foster A (2006). Trachoma control: a guide for programme managers.

[28] (2014). Solomon Islands National Statistical Office: Population and housing census 2009.

[29] Solomon Islands National Statistical Office (2014). Provincial profile of the 2009 population and housing census.

[30] Solomon Islands National Statistical Office: Projected population by province 2010–2025. Honiara: Solomon Islands National Statistical Office.

[31] Fiji Bureau of Statistics (2007). Census of population and housing. Labour force, employment and unemployment.

[32] Keen M, Barbara J (2016). Pacific urbanisation: changing times. http://devpolicy.org/pacific-urbanisation-changing-times-20160225/.

[33] Edwards IR, Aronson JK (2000). Adverse drug reactions: definitions, diagnosis, and management. Lancet.

[34] Evans JR, Solomon AW (2011). Antibiotics for trachoma. Cochrane Database Syst Rev.

[35] Romani L, Koroivueta J, Steer AC (2015). Scabies and impetigo prevalence and risk factors in Fiji: a national survey. PLoS Negl Trop Dis.

[36] Haar K, Romani L, Filimone R (2014). Scabies community prevalence and mass drug administration in two Fijian villages. Int J Dermatol.

[37] Heukelbach J, van Haeff E, Rump B, Wilcke T, Moura RC, Feldmeier H (2003). Parasitic skin diseases: health care-seeking in a slum in north-east Brazil. Trop Med Int Health.

[38] Wong LC, Amega B, Connors C (2001). Outcome of an interventional program for scabies in an indigenous community. Med J Aust.

[39] Kearns TM, Speare R, Cheng AC (2015). Impact of an ivermectin mass drug administration on scabies prevalence in a remote Australian Aboriginal community. PLoS Negl Trop Dis.

[40] Currie BJ, McCarthy JS (2010). Permethrin and ivermectin for scabies. N Engl J Med.

[41] Mounsey KE, Bernigaud C, Chosidow O, McCarthy JS (2016). Prospects for moxidectin as a new oral treatment for human scabies. PLoS Negl Trop Dis.

