# Correction to *Lancet Glob Health* 2018; 6: e1132–38

**DOI:** 10.1016/S2214-109X(18)30439-X

**Published:** 2018-09-21

*Romani L, Marks M, Sokana S, et al. Feasibility and safety of mass drug coadministration with azithromycin and ivermectin for the control of neglected tropical diseases: a single-arm intervention trial.* Lancet Glob Health *2018; 6: e1132–38*—The trial registration number has been corrected to ACTRN12615001199505. This correction has been made as of Sept 20, 2018.

