# Supplementary material for: Feasibility and safety of mass drug coadministration with azithromycin and ivermectin for the control of neglected tropical diseases: a single-arm intervention trial
Source: Lancet Glob Health. 2018 Sep 14;6(10):e1132–8. doi: 10.1016/S2214-109X(18)30397-8 (PMC6139784; doi:10.1016/S2214-109X(18)30397-8)
Supplement: Supplementary appendix [file mmc1.pdf]

THE LANCET
Global Health

Supplementary appendix

This appendix formed part of the original submission and has been peer reviewed. We post it as supplied by the authors.

Supplement to: Romani L, Marks M, Sokana O, et al. Feasibility and safety of mass drug coadministration with azithromycin and ivermectin for the control of neglected tropical diseases: a single-arm intervention trial. *Lancet Glob Health* 2018; **6:** e1132–38.

SUPPLEMENTARY TABLES

Supplementary Table 1.A: Azithromycin and ivermectin tablet dosing

WEIGHT	AZITHROMYCIN TABLETS (250mg Tabs)	IVERMECTIN TABLETS (3mg Tabs)
<12.5KG	POS*	Permethrin cream
12.5 – 25 KG	1	1
25 – 37.5 KG	2	2
37.5 – 50 KG	3	3
50-75 KG	4	4
>75 KG	4	5

*Azithromycin powder for oral suspension

Supplementary Table 1.B: Azithromycin powder for oral suspension dosing

WEIGHT	NUMBER OF ML
<3 KG	Check with Doctor
3-6 KG	2ml
6 -9 KG	4ml
9 -12 KG	6ml
12-15 KG	8ml
15 -18 KG	10ml
18-21 KG	12ml
21-24 KG	14ml
24 KG	16ml

Supplementary Table 1.C: Dosing for special situations

	TRACHOMA	SCABIES
Pregnancy	Azithromycin tablets	Permethrin cream
Breastfeeding	Azithromycin tablets	Permethrin cream
Child under 12.5kg	Azithromycin oral suspension	Permethrin cream
Child under 6 months	Topical tetracycline	Permethrin cream

Supplementary Table 2. Number of hospital admissions and deaths in Choiseul Province 12 months before and after MDA (MDA was conducted in September 2015)

Month	Admissions	Deaths
September 2014	106	6
October 2014	110	6
November 2014	100	3
December 2014	106	6
January 2015	141	4
February 2015	133	6
March 2015	167	7
April 2015	150	7
May 2015	126	8
June 2015	159	11
July 2015	102	5
August 2015	130	4
September 2015	165	5
October 2015	84	2
November 2015	109	5
December 2015	106	6
January 2016	187	17
February 2016	116	4
March 2016	183	7
April 2016	164	4
May 2016	232	8
June 2016	111	9
July 2016	92	4
August 2016	113	9
September 2016	105	8
